# Semi-Quantitative Detection of Borax Adulteration in Wheat Flour Based on Microwave Non-Destructive Testing and Machine Learning

**DOI:** 10.3390/foods15061107

**Published:** 2026-03-23

**Authors:** Mei Kang, Jiming Yang, Ya Ren, Xue Bai

**Affiliations:** 1 School of Electrical and Information Engineering, Jiangsu University, Zhenjiang 212013, China; kangmei@ujs.edu.cn (M.K.); jmy34344@gmail.com (J.Y.); 2 College of Food Science and Engineering, Henan University of Technology, Zhengzhou 450001, China; lishandya@gmail.com

**Keywords:** borax adulteration, wheat flour, microwave sensing, machine learning

## Abstract

The adulteration of wheat flour with borax poses a serious food safety risk, yet conventional rapid non-destructive screening methods remain limited. This study developed a machine learning-based microwave non-destructive semi-quantitative detection method for identifying borax adulteration in wheat flour. Using a proprietary microwave detection system, which acquires broadband frequency-domain amplitude attenuation and phase shift responses in the 2.5–11.5 GHz band, amplitude attenuation spectra and dimensional phase offset spectra were obtained from 155 samples prepared at three adulteration levels (0%, 0.1–0.9%, 1–5%). These samples simulated real-world adulteration scenarios. To address high-dimensionality and class imbalance, a hybrid Random Forest-Whale Optimization Algorithm (RF-WOA) was employed to synergistically optimize feature selection and model hyperparameters. Through hierarchical repeated validation and macro-level metric evaluation, this approach achieved an overall classification accuracy of 94.6% and a macro F1 score of 0.95 while compressing the original 1800-dimensional feature space to approximately 200 effective features. Confusion matrix analysis indicates 100% recall for undiluted samples, with misclassifications primarily occurring between adjacent adulteration levels and no false negatives introduced for adulterated samples. These results demonstrate that microwave sensing combined with the RF-WOA provides a rapid, non-destructive, and robust preliminary screening and grading evaluation strategy for borax adulteration in wheat flour, exhibiting significant potential in food safety monitoring and regulatory inspection.

## 1. Introduction

As a fundamental ingredient in numerous food products, the authenticity and safety of wheat flour directly influence public health and consumer confidence [1]. Among various forms of food adulteration, the illegal addition of borax (sodium borate) remains a persistent concern. Although borax can enhance product texture, improve shelf-life stability, and exert preservative effects, its use as a food additive is strictly prohibited under China’s Food Additive Usage Standards (GB 2760-2024) [2] and U.S. FDA regulations. Nevertheless, driven by economic incentives and compounded in some cases by environmental contamination, such practices continue to occur. Food safety investigations and case reports have documented the intentional addition of trace amounts of borax to flour-based products and meat items (e.g., noodles and meatballs) to evade routine inspections while achieving mild preservative or textural enhancements [3,4].

Toxicological evidence indicates that borax exhibits cumulative toxicity. Acute oral ingestion at doses of approximately 2–3 g may result in severe gastrointestinal irritation, renal impairment, and neurological symptoms. Moreover, prolonged exposure to trace levels may lead to chronic health hazards, including potential organ damage [3,5]. Notably, both toxicological findings and food safety investigations suggest that health risks and adulteration intent vary across different concentration ranges, thereby providing a scientific rationale for stratified contamination assessment [6]. The persistence of sporadic violations underscores the urgent need to develop rapid, reliable, and easily deployable detection technologies suitable for routine monitoring and on-site screening [7].

Traditional borax detection methods—including titration, spectrophotometry, ion chromatography, and other laboratory chemical assays—offer high sensitivity and accuracy but typically require destructive sampling, specialized operators, and time-consuming sample preparation [8,9,10,11]. These limitations severely hinder high-throughput monitoring, making frequent testing in production lines, warehouses, and market surveillance scenarios difficult to implement [12]. Consequently, non-destructive testing (NDT) methods are gaining increasing attention, as this technology enables rapid, low-cost, and minimally invasive assessment of flour adulteration without complex pretreatment [13].

Microwave non-destructive testing (MNDT) has emerged as a promising solution for food quality assessment due to its penetration capability and sensitivity to material dielectric properties [14]. Changes in moisture, ion content, and chemical composition alter dielectric constants and loss factors, thereby influencing microwave transmission, reflection, and resonance responses [15]. For powdered foods like wheat flour, borax adulteration alters electromagnetic responses by modifying ionic conductivity and bound water interactions, providing measurable physical detection evidence [16]. Compared to optical spectroscopy, microwave sensing exhibits lower sensitivity to surface scattering and offers complementary information related to bulk physicochemical properties, making it highly attractive for rapid screening applications [17]. Unlike laboratory-grade vector network analyzers (VNAs), which are costly and designed for broad-spectrum analysis, the microwave sensing system developed in this study targets a specific frequency range relevant to food adulteration detection, enabling a simpler architecture and lower cost while maintaining sufficient sensitivity for screening.

Despite these advantages, practical deployment of MNDT for adulteration control faces two methodological challenges. First, microwave measurements often yield high-dimensional feature representations (e.g., multi-frequency amplitude/phase responses), while available datasets can be relatively small due to sample preparation constraints and measurement costs [18]. Second, the regulatory and industrial need is frequently not limited to a binary decision (adulterated vs. non-adulterated) but extends to identifying contamination ranges that reflect risk levels and enforcement thresholds [19]. In this context, semi-quantitative detection—estimating adulteration severity in discrete intervals—can provide actionable information for decision-making while remaining robust to measurement variability and sample heterogeneity [20].

Machine learning (ML) offers a data-driven framework to model the complex and potentially nonlinear relationship between MNDT features and adulteration status [21]. Support vector machines (SVM) and back-propagation (BP) neural networks have been widely applied in chemometrics and sensor-based food analysis, yet their performance may degrade when the feature space is large relative to the number of samples or when hyperparameter tuning is inadequate. Random forests (RF), as an ensemble of decision trees with built-in feature subsampling and bagging, often exhibit strong generalization on small-sample, high-dimensional problems and provide a degree of robustness to noise and correlated predictors [22]. However, RF performance can still depend on the choice of feature subset and key hyperparameters (e.g., number of trees, leaf size), motivating the use of optimization strategies to improve accuracy and stability [23].

Metaheuristic optimization algorithms are increasingly used to select informative features and tune model hyperparameters in food sensing studies [24]. Among them, the whale optimization algorithm (WOA) is a population-based method inspired by the bubble-net feeding behavior of humpback whales, offering a simple structure and competitive global search capability [25]. By jointly optimizing feature selection and RF hyperparameters, the WOA has the potential to enhance discriminative performance while controlling model complexity [26]. To provide a meaningful benchmark, variable combination population analysis (VCPA) can be used as a representative feature-selection approach in high-dimensional settings, allowing for a direct comparison of optimization-driven feature selection strategies [27].

Against this backdrop, this study aims to develop a rapid, non-destructive method for detecting borax adulteration in wheat flour by integrating microwave non-destructive testing technology with machine learning techniques. By capturing broadband microwave transmission responses—including amplitude attenuation spectra and dimensional phase offset spectra—from samples, the method identifies dielectric property changes induced by borax addition. To meet practical food safety testing requirements—specifically detecting trace amounts of borax adulteration—adulterated samples were categorized into three semi-quantitative levels (0%, 0.1–0.9%, and 1–5%). This classification system accurately reflects common scenarios of illegal adulteration in regulatory monitoring and aligns with most countries’ zero-tolerance policies for borax contamination in food [28].

Given the high dimensionality of microwave features and the imbalance among contamination classes, a hybrid random forest–whale optimization algorithm (RF–WOA) was employed to jointly optimize feature selection and model hyperparameters, with the aim of improving model robustness and parsimony. Specifically, this study (1) develops a broadband microwave sensing system for non-destructive acquisition of amplitude attenuation and phase shift responses, (2) jointly exploits multi-frequency amplitude and phase information for semi-quantitative modeling, (3) introduces an RF–WOA framework for feature selection and hyperparameter optimization under class imbalance, and (4) enables graded assessment of borax adulteration severity to support food safety screening and regulatory inspection.

## 2. Materials and Methods

### 2.1. Sample Preparation and Acquisition

The wheat flour was provided by Wu deli Flour Group Co., Ltd. (Handan, China). According to the manufacturer’s nutritional label, the composition per 100 g is energy 1463 kJ, protein 9.6 g, fat 1.6 g, carbohydrates 73.0 g, and sodium 0 mg, complying with the Chinese national standard for wheat flour (GB/T 1355-2021) [29]. Borax (sodium tetraborate) was purchased from Sinopharm Chemical Reagent Co., Ltd. (Shanghai, China) and met the requirements of GB/T 632-2008 [30].

Experimental samples prepared by mixing borax with wheat flour were portioned into 50-mL centrifuge tubes to form dry solid mixtures based on mass ratios. Wheat flour and borax powder were added to each tube according to target mass fractions, then mixed for one minute using a centrifugal homogenizer (Super MiniMax 10k, ANPRO Biotechnology Co., Ltd., Chengdu, China). To enhance uniformity and prevent overheating, the mixing operation was repeated three times with 30-s intervals between each cycle. To ensure samples remained in a dry powder state throughout, all processing and measurements were conducted under controlled laboratory conditions (temperature 23 ± 1 °C, relative humidity 50 ± 5%). A total of 155 samples were prepared, each contained in a dedicated testing container. Based on actual illicit usage scenarios, adulteration levels were categorized into three groups: no adulteration (0%, n = 13), low-level adulteration (0.1–0.9%, n = 41), and high-level adulteration (1–5%, n = 101).

### 2.2. Microwave Sensing System and Frequency-Response Acquisition

**Figure 1:**
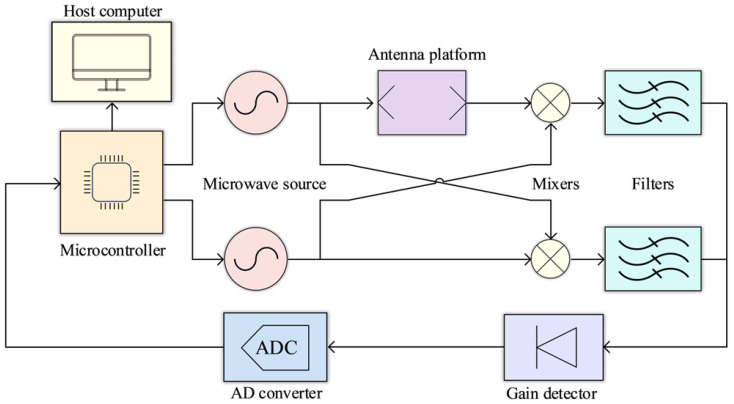
A self-designed microwave detection device.

**Figure 2:**
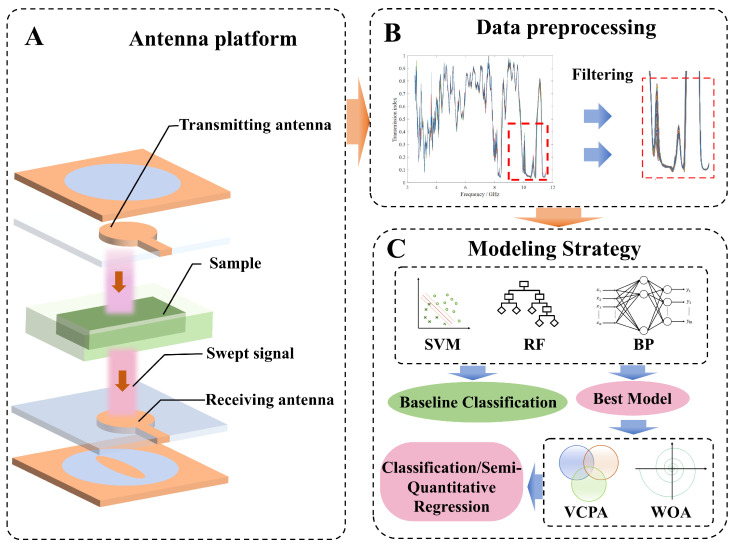
Technical framework of the prediction model research. (**A**) Antenna platform. (**B**) Data preprocessing. (**C**) Modeling Strategy. In the SVM diagram, colored lines represent decision boundaries and margins, circles and crosses denote different sample categories, and arrows indicate the data processing or classification direction.

The proprietary microwave sensing system shown in Figure 1 acquires the frequency-domain electromagnetic response of wheat flour samples across a wide bandwidth range. Controlled by an STM32F407VGT6 microcontroller (STMicroelectronics, Geneva, Switzerland), the system incorporates two microwave sources. One source generates a swept signal from 2.5 to 11.5 GHz with 10 MHz steps (covering 900 frequency points), while the other source provides a 1 GHz continuous-wave reference signal. The microwave platform is illustrated in Figure 2A.

During measurement, the swept signal was transmitted through the flour sample using an antenna-based transmission configuration. As microwaves propagate through heterogeneous materials, the transmitted signal undergoes multiple effects, primarily amplitude attenuation and phase shift, which are governed by the dielectric properties and are influenced by material composition, density, and microstructure. Because wheat flour and borax differ in physicochemical properties, borax adulteration produces distinguishable patterns of attenuation and phase shift across the 2.5–11.5 GHz band.

To extract the sample-induced response, the transmitted swept signal was mixed with the fixed-frequency reference signal and compared against a baseline signal acquired under empty (no-sample) conditions. The resulting amplitude and phase information was converted into voltage outputs by an amplitude/phase detector chip. The voltage signals were digitized by an analog-to-digital converter (ADC) and sent to the STM32 microcontroller, then transferred via serial communication to a host computer for visualization (imaging), storage, and subsequent analysis.

For each of the 155 samples, the system produced two 900-dimensional vectors: an amplitude attenuation spectrum and a phase shift spectrum. These were concatenated at the data level to form a single 1800-dimensional feature vector for modeling. To mitigate the effects of instrument noise, temperature fluctuations, and operator-related variability, the raw microwave sequences were preprocessed using a window size 5 least-squares smoothing method, as shown in Figure 2B. This preprocessing was applied to both the attenuation and phase sequences prior to multivariate modeling. After filtering, the two modalities were concatenated to obtain the final 1800-dimensional feature vector per sample (with a detection cycle of 60 s).

### 2.3. Chemical Method Reference Validation Analysis

To verify the presence of borax adulteration in prepared wheat flour samples, a chemical validation method based on curcumin UV-visible spectrophotometry was employed. This chemical analysis was used to obtain borax adulteration results in wheat flour and provide a reference basis for evaluating the microwave detection method. The chemical analysis procedure is detailed as follows: Take 1.000 g of the selected flour sample, add 20.0 mL of distilled water, mix with a vortex mixer for 1 min, then shake at room temperature for 10 min. Centrifuge the suspension at 5000 rpm for 10 min and collect the supernatant for analysis. Color development step: Mix 2.0 mL of the extract with 2.0 mL of 0.1% curcumin ethanol solution and incubate at 50 °C for 10 min. After cooling to room temperature, measure the absorbance at 540 nm using a UV-Vis spectrophotometer (UV-2600, Shimadzu, Kyoto, Japan) with a 1 cm path length. Nine representative samples containing known concentrations of borax were selected for chemical analysis. Each sample underwent three replicate tests, with the average value serving as the borax concentration determined by the approved chemical method. Based on the contamination levels defined in this study, chemically measured concentrations were further categorized into three groups: no adulteration (0%), low-level adulteration (0.1–0.9%), and high-level adulteration (1–5%). These chemical classifications served as reference labels for evaluating the microwave non-destructive testing model.

### 2.4. Dataset Construction and Sample Partitioning

Based on toxicological thresholds and documented illicit usage patterns, this study categorizes samples into three contamination tiers: 0% (no addition), 0.1–0.9% (low-level adulteration, simulating covert and difficult-to-detect illegal use), and 1–5% (high-level adulteration, simulating deliberate excessive addition). This stratified design enables the model to assess sensitivity to micro-level hazards posing significant health risks while maintaining robustness under overt illegal adulteration scenarios, thereby enhancing the practical relevance of the proposed detection framework.

Given the substantial class imbalance (13/41/101), a repeated stratified random sub-sampling validation strategy was adopted to obtain robust and unbiased performance estimates while strictly preserving the original class distribution. Specifically, the dataset was first stratified according to class labels, and random sampling was then independently conducted within each stratum to partition the samples into a training subset and a test subset, with approximately 70% of samples allocated for training and the remaining 30% reserved for testing, thereby ensuring consistent class proportions across subsets. In each repetition, all procedures including model training, feature selection, and hyperparameter optimization were performed exclusively within the training subset, while the test subset was strictly reserved for final performance evaluation, effectively preventing any information leakage. This entire stratification–sampling–training–testing process was repeated 20 times using different random seeds to comprehensively assess model stability and to quantify the performance variability induced by data partitioning.

### 2.5. Semi-Quantitative Modeling Strategy

**Figure 3:**
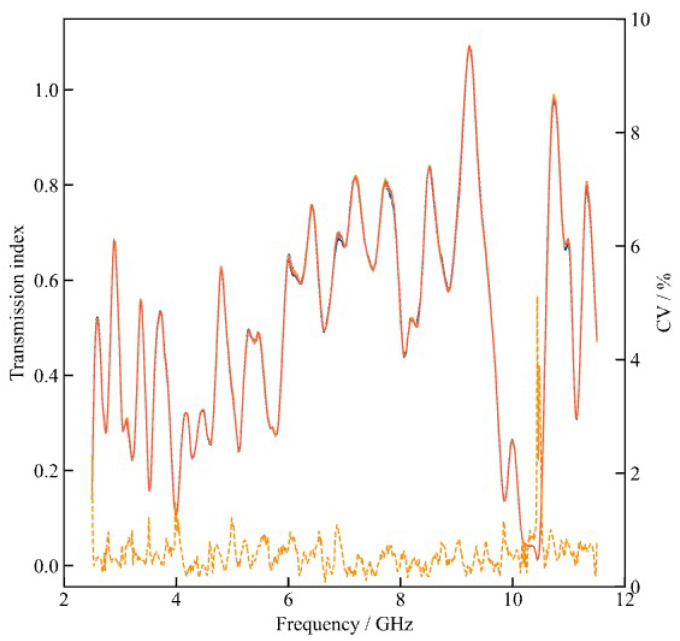
Dielectric response curves of five different batches of wheat flour. The solid red line represents the transmission index, while the dashed orange line represents the coefficient of variation (CV).

To avoid potential interference from wheat flour composition and quality in borax adulteration detection, this study selected five different batches of wheat flour. The flour was homogenized for one minute using a mixer and then sieved through a 90-mesh screen. A 2 ± 0.01 g sample from each batch was placed into the testing apparatus. The resulting five sets of sample curves are shown in Figure 3. No significant differences were observed between the curves of the different sample groups. Furthermore, in food safety testing, the primary analytical objective is often not the precise determination of contaminant concentration but rather the reliable differentiation of contamination severity and associated risk levels [31]. Under these conditions, strict quantitative regression may impose unrealistic assumptions regarding linear relationships, measurement precision, and concentration continuity. This study employs a semi-quantitative (ordinal) modeling strategy positioned between precise quantitative regression and nominal classification [32]. This model does not predict exact borax concentrations but identifies ordered contamination levels, preserving severity rankings while reducing sensitivity to uncertainties in reference measurements.

Specifically, borax contamination levels were categorized into three ordinal groups based on regulatory relevance and practical risk differentiation: uncontaminated samples (0%), low-level contamination (0.1–0.9%), and high-level contamination (1–5%). These categories are encoded as ordinal labels 0, 1, and 2. This coding explicitly preserves the natural ordering among contamination levels while avoiding assumptions about linear distribution of concentration intervals. Based on this semi-quantitative framework, a classification model was developed to distinguish contamination levels. Beyond conventional classification accuracy, the random forest-based model was evaluated using an ordinal-aware performance metric that reflects both the magnitude and direction of misclassification. This approach not only assesses prediction correctness but also determines whether misclassifications occur between adjacent or distant contamination levels, providing more informative model reliability assessment for practical risk evaluation scenarios.

### 2.6. Baseline Classification Models

To establish performance benchmarks and evaluate the discriminative capability of microwave features under different learning paradigms, three representative classifiers were selected as reference models, as shown in Figure 2C: support vector machine (SVM), backpropagation neural network (BP), and random forest (RF) [31,32,33,34]. These baselines cover complementary modeling philosophies, including kernel-based learning (SVM), shallow neural learning (BP), and ensemble learning (RF), and provide a fair benchmark prior to introducing feature selection and metaheuristic optimization. All baseline models were trained using the full 1800-dimensional feature vectors (after smoothing) without additional feature selection or hyperparameter optimization beyond standard settings.

The Support Vector Machine (SVM) classifier employs a Gaussian radial basis function (RBF) kernel and adopts a one-versus-one strategy for three-class classification in MATLAB (MathWorks, Natick, MA, USA, R2023b) [35]. Prior to model training, a lightweight variance-based feature preselection is conducted using only the training data. Feature variances are calculated and ranked, and the top-ranked features are retained as inputs. Rather than serving as an independent feature selection algorithm, this procedure functions as a computationally efficient noise-reduction and dimensionality-control strategy, mitigating the adverse effects of redundant or low-information variables within the original 1800-dimensional feature space.

The Shallow Neural Learning model is implemented as a single-hidden-layer feedforward neural network trained using a standard backpropagation framework [36]. A compact network architecture is adopted by limiting the number of hidden-layer neurons, and an early-stopping strategy based on validation performance is applied [37]. During training, the available data are internally partitioned into training, validation, and monitoring subsets, and learning is terminated when validation performance fails to improve over consecutive iterations. This approach effectively constrains model complexity and reduces the risk of overfitting. To further enhance training stability, the number of iterations is restricted, and all preprocessing procedures, including normalization, are performed exclusively on the training data to prevent information leakage.

The baseline RF configuration constructs 25 trees with a minimum leaf size of 8 to prevent excessive tree depth. Random subsampling of predictor variables during each split effectively controls model complexity and inter-tree correlation. This configuration balances discriminative power, computational efficiency, and generalization stability.

### 2.7. Feature Selection and RF Optimization Frameworks

#### 2.7.1. Proposed RF–WOA Joint Optimization Framework

This paper proposes a hybrid Random Forest-Whale Optimization Algorithm (RF-WOA) framework that collaboratively optimizes feature selection and Random Forest hyperparameters within a unified envelope optimization scheme. By processing feature selection and model optimization in series, the two processes are encoded as a single candidate solution [38]. Specifically, each whale individual represents a solution vector z=[b,T,L], where $b\in {\left[0,1\right]}^{D}$ is a continuous feature selection vector, *T* denotes the number of trees in the RF model, and *L* represents the minimum leaf size. The continuous feature vector *b* is converted into a binary feature mask through a rule that partitions it based on distinguishing the magnitude of the $b_{i}$ threshold values, where at least one feature is mandatorily retained to prevent degeneracy. The RF hyperparameters are searched within predefined ranges, with T∈[10,60] and L∈[5,20].

The optimization objective is to maximize stratified five-fold cross-validated classification accuracy on the training subset while simultaneously discouraging overly large feature subsets. Accordingly, the fitness function is defined as

(1) $$\mathrm{Score}={\mathrm{Acc}}_{\mathrm{CV}}-\lambda \cdot \frac{k}{D},$$

where ${\mathrm{Acc}}_{\mathrm{CV}}$ denotes the cross-validated accuracy, *k* is the number of selected features, D=1800 is the total feature dimension, and λ=0.003 controls the trade-off between classification performance and feature sparsity. All fitness evaluations are conducted exclusively on the training data to prevent information leakage.

Compared with commonly used optimizers such as PSO and GA, the whale optimization algorithm (WOA) offers a balanced exploration–exploitation mechanism that helps mitigate premature convergence in high-dimensional feature spaces. This characteristic makes it well suited for jointly optimizing feature selection and RF hyperparameters in the 1800-dimensional microwave sensing dataset. The whale optimization algorithm is implemented with a population size of 18 and 80 iterations, following standard encircling, bubble-net attacking, and search-for-prey mechanisms with appropriate boundary-handling strategies. Through iterative updates, RF–WOA explores the joint feature–hyperparameter space and converges toward solutions that balance predictive accuracy and model compactness. After optimization, the RF classifier is retrained on the full training subset using the selected feature subset and optimized hyperparameters obtained from the best whale individual. The resulting model is then evaluated on the corresponding held-out test subset. Detailed parameter settings and search ranges are provided in Table A1 to ensure transparency and reproducibility.

#### 2.7.2. Proposed RF–VCPA Joint Optimization Framework

A comparative sequence feature selection and modeling framework based on Variable Combination Population Analysis (VCPA), termed RF-VCPA, employs a two-stage process: first performing independent feature selection, followed by training a Random Forest (RF) model on the refined feature subset. Variable Combination Population Analysis (VCPA) is a wrapper-based random feature selection method that iteratively identifies informative variables by evaluating populations of randomly generated feature subsets and progressively narrowing the candidate space [39]. Variable combination population analysis (VCPA) is a stochastic, wrapper-based feature selection method that iteratively identifies informative variables by evaluating populations of randomly generated feature subsets and progressively shrinking the candidate space. Let $X\in R^{N\times D}$ denote the original feature matrix, where *N* is the number of samples and D=1800 is the total number of microwave features.

At iteration *t*, VCPA maintains a candidate feature pool $F^{\left(t\right)}\subseteq \left\{1,2,\ldots ,D\right\}$, $|F^{\left(t\right)}|=d_{t}$. An initial retained ratio $r_{0}=0.25$ was used, yielding $d_{0}=\left\lfloor r_{0}\cdot D\right\rfloor =450$. For each iteration, a population of P=18 candidate feature subsets is randomly generated by sampling combinations from $F^{\left(t\right)}$. Each candidate subset is evaluated using a random forest classifier trained on the training subset, and its performance is quantified by stratified cross-validated classification accuracy:

(2) $${\mathrm{Acc}}_{\mathrm{CV}}^{\left(i\right)}=\mathrm{RF}\text{-}\mathrm{CV}\left(X_{S_{i}},y\right),\;i=1,\ldots ,P,$$

where $S_{i}\subseteq F^{\left(t\right)}$ denotes the *i*-th feature combination.

After evaluating the population, the top fraction ρ=0.25 of candidate subsets with the highest accuracies is retained. Feature selection frequencies are then updated based on their occurrence within these top-performing subsets. It can be expressed by the following formula:

(3) $$f_{j}^{\left(t\right)}=\sum_{i\in T}I\left(j\in S_{i}\right),$$

where T denotes the index set of the top-performing candidates and $I(j\in S_{i})$ is the indicator function. The candidate feature pool is subsequently reduced using a shrink factor α=0.85, such that $d_{t+1}=\left[\alpha \cdot d_{t}\right]$. The $d_{t+1}$ features with the highest frequencies $f_{j}^{\left(t\right)}$ are retained to form the updated pool $F^{\left(t+1\right)}$. This iterative process continues for a maximum of 35 iterations or until no further improvement in validation accuracy is observed.

Once VCPA converges, the final selected feature subset $F^{*}$ is used to train an RF classifier. To ensure a fair comparison with the RF–WOA framework, RF hyperparameters were tuned within constrained ranges rather than jointly optimized. Specifically, the number of trees was searched within [10,30], and the minimum leaf size within [5,10], from which the best-performing configuration (*Trees* =30, *MinLeafSize* =10) was consistently obtained across runs. The parameter mtry, representing the number of predictors randomly sampled at each split, was set adaptively according to the dimensionality of the selected feature subset $p_{\mathrm{sel}}=\left|F^{*}\right|$, following the default RF heuristic to maintain comparable model capacity across different feature set sizes.

RF–VCPA training and evaluation procedures were conducted exclusively on the training subset during feature selection to prevent information leakage, and final performance was assessed on the held-out test subset. Unlike RF–WOA, where feature selection and RF hyperparameters are encoded into a single optimization vector and jointly optimized under a unified objective function, RF–VCPA decouples these two processes. While this sequential strategy often yields highly compact feature subsets, it may overlook interactions between feature selection and classifier configuration, potentially limiting its ability to achieve globally optimal solutions under high-dimensional and class-imbalanced conditions.

### 2.8. Evaluation Metrics

Classification performance was primarily evaluated using overall accuracy (OA), together with macro-averaged precision (Macro-P), macro-averaged recall (Macro-R), and macro-averaged F1-score (Macro-F1), to provide a balanced assessment under the imbalanced three-class setting [40]. Higher OA indicates stronger overall classification capability, while higher Macro-P, Macro-R, and Macro-F1 reflect improved average class-wise performance and a better balance between misclassification and omission errors across classes. In addition, per-class precision, recall, F1-score, and confusion matrices were analyzed to characterize class-specific classification behavior.

Under the semi-quantitative framework, model performance was evaluated using root mean square error (RMSE), mean absolute error (MAE), and the coefficient of determination (R^2^), which quantify the deviation between predicted and reference ordinal levels. Lower RMSE and MAE values indicate smaller ordinal prediction errors, reflecting higher consistency with the reference contamination grades, while higher R^2^ values indicate stronger agreement in the overall ordinal trend. These semi-quantitative regression results were jointly interpreted with classification performance to provide a comprehensive evaluation of model effectiveness for food safety screening and contamination risk-level assessment.

All classification metrics were summarized as mean ± standard deviation over 20 repeated stratified random splits to evaluate both predictive accuracy and model stability. To statistically compare RF–WOA with baseline RF and RF–VCPA models, paired *t*-tests and Wilcoxon signed-rank tests were conducted on paired results obtained from repeated runs, with statistical significance assessed at a 95% confidence level (α = 0.05).

## 3. Results and Discussion

### 3.1. Baseline Classification Performance

The classification performance of the baseline models is summarized in Table 1. Among the three classifiers, the Support Vector Machine (SVM) demonstrated the weakest discrimination capability, achieving an average overall accuracy (OA) of 76.8%. Similar observations have been reported in spectroscopic adulteration detection studies, where SVM often shows limited discrimination capability when dealing with high-dimensional spectral data compared with ensemble learning models such as Random Forest [41]. Both its macro-average recall (0.71) and macro F1 score (0.69) remained at relatively low levels, indicating that SVM struggles to distinguish subtle dielectric differences between adjacent borax adulteration levels when processing high-dimensional microwave features. The backpropagation neural network (BP) achieved higher mean accuracy (90.4%) and macro F1 score (0.91), but its performance exhibited significant fluctuations across repeated runs, evidenced by high standard deviation. This instability indicates susceptibility to random initialization and data partitioning, limiting its robustness for trace adulteration detection. In contrast, the Random Forest (RF) classifier consistently demonstrated superior and balanced performance, achieving an overall accuracy of 93.9% and a macro F1 score of 0.94. Its high macro precision and macro recall confirm RF’s ability to reliably distinguish between all three contamination levels. These results validate RF as the optimal baseline model and justify its selection as the foundation for subsequent optimization of base learners.

### 3.2. Performance of Optimized RF-Based Models

#### 3.2.1. Convergence Behavior of Optimization Algorithms

**Figure 4:**
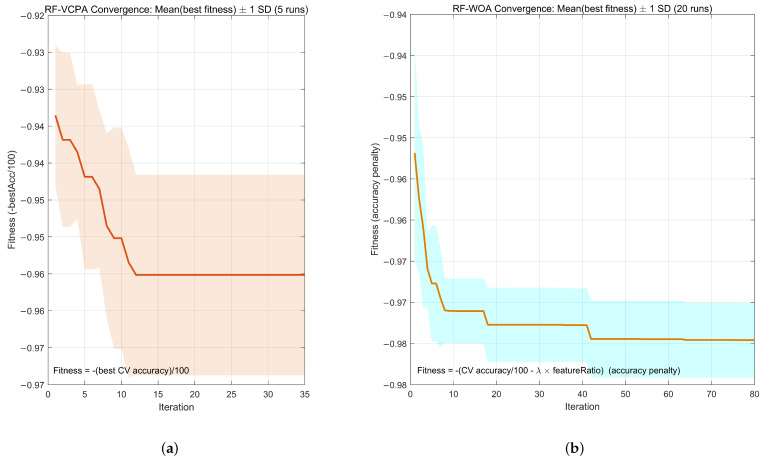
Compare the convergence behavior of the two models. (**a**) Convergence curve (20 runs) RF-VCPA. (**b**) Convergence curve (20 runs) RF-WOA. The solid line represents the mean best fitness value, while the shaded background indicates ±1 standard deviation across multiple runs.

Figure 4a and Figure 4b illustrate the convergence characteristics of RF–WOA and RF–VCPA, respectively. As shown in Figure 4a, RF–WOA exhibits rapid convergence in early iterations, with the fitness value stabilizing within approximately 10–15 iterations. The shaded region (representing standard deviation across 20 runs) remains consistently narrow, indicating strong stability and robustness of the RF–WOA. This validates the effectiveness of WOA in jointly optimizing feature selection and RF hyperparameters. Figure 4b shows that RF–VCPA converges more slowly with noticeable fluctuations during early iterations. Although it eventually converges, the larger amplitude of fluctuations indicates greater sensitivity to feature subset initialization. This difference explains why RF–WOA achieves slightly superior and more stable classification performance compared to RF–VCPA.

#### 3.2.2. Classification Performance and Feature Reduction

Table 2 summarizes the classification performance of the proposed RF–WOA model across 20 runs of stratified holdout set cross-validation. Under identical data partitioning conditions, the RF–WOA model achieved an average overall accuracy of 94.6% and a macro F1 score of 0.95, slightly outperforming the baseline RF model. Related spectral detection methods have also reported comparable accuracy. For instance, near-infrared spectroscopy combined with chemometric models achieved a coefficient of determination exceeding 0.97 in detecting illicit additives in wheat flour [41]. However, this approach requires complex optical instruments and is more susceptible to surface scattering effects. In contrast, hyperspectral imaging methods typically attain prediction accuracies ranging from 0.85 to 0.90, generally lower than the prediction accuracy achieved in this study [42]. Paired two-tailed *t*-tests and Wilcoxon signed-rank tests (Table A2) indicate that the difference in macro F1 scores between RF–WOA and RF models is not statistically significant (*p*-values of 0.21 and 0.24, respectively). However, RF–WOA demonstrates a clear advantage in feature efficiency, This algorithm selected an average of 200 features from the original 1800-dimensional microwave feature space, achieving nearly 90% dimensionality reduction while maintaining robust and reliable prediction performance. Such significant feature dimension compression demonstrates RF–WOA’s ability to identify compact and information-rich feature subsets without sacrificing classification accuracy. In contrast, the RF–VCPA model, independent of hyperparameter optimization, exhibited poorer classification performance (overall accuracy 91.5%, macro F1 score 0.91). Moreover, it selected 140 features across multiple runs, indicating high sensitivity to data partitioning and low feature selection stability. Notably, paired statistical tests reveal that RF–WOA significantly outperforms RF–VCPA in macro F1 scores for classification tasks (paired *t*-test *p* = 0.031; Wilcoxon signed-rank test *p* = 0.041; see Table A2). These results demonstrate that by jointly optimizing feature selection and model hyperparameters, RF–WOA enhances both classification performance and stability compared to sequential feature selection strategies. Overall, the core advantage of the RF–WOA framework lies in boosting model robustness, stability, and interpretability, rather than merely pursuing marginal statistical improvements in mean accuracy.

#### 3.2.3. Confusion Matrix Analysis

A confusion matrix is a class-wise contingency table that summarizes the correspondence between predicted labels and true labels for a multi-class classification task. Based on the confusion matrix, class-wise recall and precision are computed as derived performance metrics. Recall measures the ability of the classifier to correctly identify samples belonging to a given class and is calculated in a row-wise manner as ${\mathrm{Recall}}_{i}=\frac{TP_{i}}{TP_{i}+FN_{i}}$, where $TP_{i}=C_{ii}$ denotes the number of correctly classified samples of class *i*, and $FN_{i}=\sum_{j\neq i}C_{ij}$ represents the samples of class *i* misclassified as other classes. Precision evaluates the reliability of predicted class assignments and is computed column-wise as ${\mathrm{Precision}}_{i}=\frac{TP_{i}}{TP_{i}+FP_{i}}$, where $FP_{i}=\sum_{j\neq i}C_{ji}$ denotes the number of samples incorrectly predicted as class *i*.

**Figure 5:**
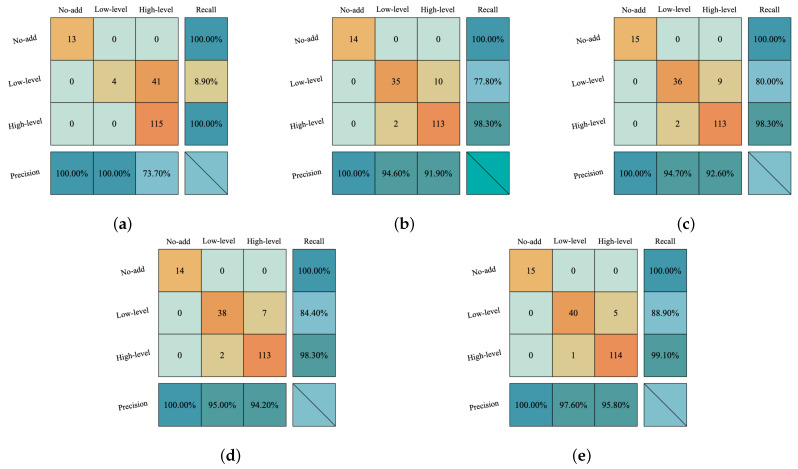
Confusion matrices obtained by different methods. (**a**) SVM confusion matrix. (**b**) BP confusion matrix. (**c**) RF confusion matrix. (**d**) RF-VCPA confusion matrix. (**e**) RF-WOA confusion matrix. The color intensity represents the number of samples, where darker colors indicate higher values. Correct classifications are mainly concentrated along the diagonal, while off-diagonal elements represent misclassifications.

Figure 5 displays the aggregated confusion matrices for the baseline SVM, BP, and RF models. The SVM model exhibits a distinct pattern of classification errors: unadulterated samples (0%) are typically correctly identified, but a large number of low-adulteration samples (0.1–0.9%) are misclassified as high-adulteration samples (1–5%). This results in significantly reduced recall for low-level adulteration categories, as the SVM’s linear decision boundary struggles to distinguish adjacent contamination levels in the high-dimensional microwave feature space, obscuring the gradual transitions between contamination severity. The BP neural network significantly improved recall for adulterated samples, but its confusion matrix revealed substantial variability across categories, occasionally mixing unadulterated and adulterated samples. This instability indicates sensitivity to random initialization and limited robustness when trained on relatively small datasets. The Random Forest benchmark model maintains stable and excellent classification performance across all categories. As shown in Figure 5c, the vast majority of samples are correctly classified, with misclassifications primarily occurring between adjacent adulteration levels. Confusion between unadulterated and adulterated samples is extremely rare, reflecting the Random Forest classifier’s robust nonlinear modeling capabilities and ensemble stability.

The RF-VCPA confusion matrix in Figure 5d demonstrates that aggressive feature selection can significantly reduce feature dimensions while substantially preserving classification performance. The model maintains high recall for uncontaminated samples and acceptable identification rates for contaminated ones. However, compared to the full RF model, the misclassification rate between low and high contamination levels slightly increases. This indicates that while VCPA effectively removes redundant variables, excessive feature dimensionality reduction may lose feature information crucial for distinguishing between adjacent contamination levels. Figure 5e displays the aggregated confusion matrix after 20 iterations of the RF–WOA model. Since this matrix is derived from test predictions across all iterations, the total sample count exceeds the number of unique samples, representing the combined test set. RF–WOA achieves perfect identification (0% misclassification rate) for unadulterated samples with 100% recall. For low-level adulteration (0.1–0.9%) samples, the correct classification rate is 88.9%, with 11.1% misclassified as high-level adulteration (1–5%). The recall rate for high-level adulterated samples reached 99.1%, with only 0.9% misclassified as low-level contamination. Misclassification between undiluted and adulterated samples was extremely rare, with most errors occurring between adjacent adulteration levels—consistent with the ordinal nature of semi-quantitative tasks. From a food safety perspective, false negatives (adulterated samples misclassified as pure) represent the most critical error because they may allow contaminated products to pass screening undetected. In this study, such cases were extremely rare, with misclassifications mainly occurring between adjacent adulteration levels. Table 3 summarizes the RF-WOA model’s class accuracy, recall rate, and F1 score. Notably, the method achieved 100% recall for unadulterated samples, with no unadulterated samples misclassified as adulterated, demonstrating its safety-oriented reliability.

### 3.3. Semi-Quantitative Regression Results

In addition to discrete classification, semi-quantitative regression was employed to directly estimate borax concentration (%). The semi-regression results are summarized in Table 4, with data based on 20 random stratified runs and reported as mean ± standard deviation. Support vector machines demonstrated the poorest performance (root mean square error =0.24±0.04; mean absolute error =0.12±0.04; $R^{2}=0.41\pm 0.03$), indicating weak correlation between predicted and standard values and limiting the practical value of semi-quantitative estimation. The BP model significantly improved accuracy ($R^{2}=0.83\pm 0.11$; RMSE =0.09±0.07), but its high variability indicates sensitivity to data partitioning and initialization settings, consistent with the instability observed in classification analyses. Among baseline models, Random Forest (RF) demonstrated the most precise and stable regression performance (RMSE =0.07±0.03; MAE =0.07±0.03; $R^{2}=0.84\pm 0.08$), reflecting robust nonlinear modeling capability and ensemble stability. After optimization, the RF–WOA model achieved the best overall regression results (RMSE =0.06±0.03; MAE =0.06±0.03; $R^{2}=0.86\pm 0.07$), while the RF–VCPA model exhibited a moderate trade-off between model compactness and numerical accuracy ($R^{2}=0.78\pm 0.10$). Previous studies have demonstrated that hyperspectral imaging methods typically achieve R^2^ values ranging from 0.80 to 0.90 in grain quality assessment. Although the R^2^ values obtained in this study are slightly lower than those achieved by certain optical spectral methods, the proposed microwave sensing framework exhibits superior applicability and stability, enabling non-destructive rapid detection without the need for optical scanning systems [42,43]. Pairwise statistical comparisons based on 20 replicates of stratified holdout sets (Table A2) indicate that the RF–WOA model outperforms the baseline RF model on semi-quantitative regression metrics. Both paired *t*-tests and Wilcoxon signed-rank tests yielded *p* > 0.05, suggesting that the observed improvement, while incremental, is robust. RF–WOA significantly outperformed RF–VCPA across all semi-quantitative regression metrics, with both parametric and nonparametric tests revealing statistically significant differences (*p* ≤ 0.01). These findings demonstrate that combining feature selection via WOA with hyperparameter optimization yields a more effective and stable modeling strategy compared to sequential feature selection methods. Thus, the RF–WOA model achieves a favorable balance between predictive accuracy, robustness, and feature efficiency, making it highly suitable for rapid non-destructive screening and risk grading assessment of borax-adulterated wheat flour.

### 3.4. Verification of Microwave Semi-Quantitative Regression Method for Chemical Analysis

To evaluate the reliability of the proposed microwave detection framework, the optimal model (RF-WOA) was employed to predict borax contamination levels in wheat flour samples based on microwave dielectric measurements. The classification results obtained from the microwave model were compared with contamination levels determined by chemical analysis. As shown in Table 5, the contamination levels predicted by the microwave model generally aligned with the classification results from chemical analysis. Within the tested concentration range, the vast majority of samples were correctly classified, with only one set of samples from a borderline region being identified as highly adulterated. The overall classification accuracy reached 92.3%, indicating high consistency between the microwave detection framework and the certified chemical method. These results demonstrate that microwave sensing technology combined with the RF-WOA model can effectively identify borax contamination levels in wheat flour samples, serving as a rapid, non-destructive testing tool for food safety inspection. Traditional chemical methods typically require complex sample preparation and time-consuming analysis. Therefore, microwave detection combined with machine learning offers the potential for rapid food safety testing [44,45].

## 4. Conclusions

This study developed a machine learning model architecture integrating random forest and whale optimization algorithms based on microwave non-destructive testing technology, enabling rapid detection and semi-quantitative assessment of borax adulteration in wheat flour. Under configured experimental temperature and humidity conditions, the RF-WOA model achieved optimal predictive performance within the selected microwave frequency range using sample boxes. By integrating 2.5–11.5 GHz microwave frequency response measurements with multivariate modeling, this approach enables non-destructive differentiation of varying adulteration levels under real-world imbalanced classification conditions. The hybrid Random Forest-Whale Optimization Algorithm (RF-WOA) enhances model stability and robustness by integrating feature selection with hyperparameter optimization, significantly reducing dimensionality while maintaining reliable classification performance. Confusion matrices and semi-quantitative regression analysis demonstrate stable model performance, with misclassifications primarily occurring between adjacent adulteration levels and extremely low false-negative risks in adulterated samples. Overall, this framework provides a rapid, cost-effective, and stable preliminary screening strategy for borax adulteration in wheat flour. The limitations of this study arise from the fact that all experiments were conducted under controlled laboratory conditions, with relatively limited sample diversity and size. Future research will focus on validation using larger and more diverse sample sets, evaluating multiple types of adulterants, and further optimizing the system for portable and field-deployable applications.

## 5. Collaborative Basis

Jiangsu University and Henan University of Technology jointly conducted collaborative research on semi-quantitative detection of borax adulteration in wheat flour using microwave non-destructive testing and machine learning technologies. Henan University of Technology provided test samples and experimental guidance for the borax-adulterated wheat flour experiments in this paper. Jiangsu University was responsible for designing the microwave non-destructive testing apparatus, conducting the borax adulteration detection experiments, and analyzing the data.

## Figures and Tables

**Table 1:** Classification performance of baseline models on the test set (mean ± SD).

Model	OA (%)	Macro-P	Macro-R	Macro-F1
SVM	76.80±2.50	0.90±0.03	0.71±0.04	0.69±0.04
BP	90.40±7.90	0.94±0.06	0.89±0.08	0.91±0.07
**RF**	93.90±3.10	0.96±0.02	0.93±0.03	0.94±0.03

**Table 2:** Performance comparison of RF-based models on the test set (mean ± SD).

Model	OA (%)	Macro-F1	Selected Features	Key Hyperparameters
RF	93.90±3.10	0.94±0.03	1800	Trees = 25; Leaf = 8; mtry = 21
**RF–WOA**	94.60±2.90	0.95±0.03	200	**Optimized per run**
RF–VCPA	91.50±4.00	0.91±0.05	140	Trees = 30; Leaf = 10; mtry = 14

**Table 3:** Class-wise performance of RF–WOA on the test set.

Class	Precision	Recall	F1-Score
Class 1 (0%)	1.00	1.00	1.00
Class 2 (0.1–0.9%)	1.00	0.89	0.94
Class 3 (1–5%)	0.96	1.00	0.98

**Table 4:** Semi-quantitative regression performance of RF-based models (mean ± SD).

Model	RMSE	MAE	R^2^
SVM	0.24±0.04	0.12±0.04	0.41±0.03
BP	0.09±0.07	0.08±0.04	0.83±0.11
RF	0.07±0.03	0.07±0.03	0.84±0.08
**RF–WOA**	0.06±0.03	0.06±0.03	0.86±0.07
RF–VCPA	0.09±0.04	0.09±0.04	0.78±0.10

**Table 5:** Comparison between chemical analysis classification and RF–WOA microwave analysis results.

Sample	Chemical Concentration (%)	Chemical Class	RF–WOA
S1	0.00	No-add	No-add
S2	0.12	Low-add	Low-add
S3	0.35	Low-add	Low-add
S4	0.72	Low-add	Low-add
S5	0.95	Low-add	High-add
S6	1.25	High-add	High-add
S7	2.10	High-add	High-add
S8	3.45	High-add	High-add
S9	4.10	High-add	High-add

## Data Availability

The data presented in this study are available on request from the corresponding author due to privacy.

## Appendix A. Results of Paired Statistical Tests and Hyperparameters

**Table A1:** Search space and optimized hyperparameters for RF–WOA.

Component	Definition/Search Space	Implementation
Feature mask	1800-dim binary vector	WOA continuous → threshold 0.5
$n_{\mathrm{trees}}$	[10, 60]	WOA-optimized
Min leaf size	[5, 20]	WOA-optimized
mtry	Adaptive	$\left[{\left(\mathrm{selected}\;\mathrm{features}\right)}^{1/2}/2\right]$
Fitness	Stratified *k*-fold CV	Training set
Objective	CV accuracy + penalty	Feature-regularized accuracy

**Table A2:** Results of paired statistical tests for classification and semi-quantitative regression performance over repeated stratified hold-out runs.

Model A	Model B	Task	Metric	*p* (Paired *t*-Test)	*p* (Wilcoxon)
RF–WOA	RF	Classification	Macro-F1	0.21	0.24
RF–WOA	RF–VCPA	Classification	Macro-F1	0.03	0.04
RF–WOA	RF	Semi-quantitative regression	RMSE	0.18	0.22
RF–WOA	RF–VCPA	Semi-quantitative regression	RMSE	0.01	0.01
RF–WOA	RF	Semi-quantitative regression	MAE	0.20	0.25
RF–WOA	RF–VCPA	Semi-quantitative regression	MAE	0.01	0.01
RF–WOA	RF	Semi-quantitative regression	$R^{2}$	0.16	0.19
RF–WOA	RF–VCPA	Semi-quantitative regression	$R^{2}$	0.00	0.01
